# Buccal Thin Films as Potent Permeation Enhancers for Cytisine Transbuccal Delivery

**DOI:** 10.3390/membranes12111169

**Published:** 2022-11-21

**Authors:** Viviana De Caro, Giuseppe Angellotti, Fabio D’Agostino, Giulia Di Prima

**Affiliations:** 1 Dipartimento di Scienze e Tecnologie Biologiche Chimiche e Farmaceutiche (STEBICEF), University of Palermo, Via Archirafi 32, 90123 Palermo, Italy; 2 Dipartimento di Discipline Chirurgiche, Oncologiche e Stomatologiche (DICHIRONS), University of Palermo, Via L. Giuffrè 5, 90127 Palermo, Italy; 3 Istituto per lo Studio degli Impatti Antropici e Sostenibilità dell’Ambiente Marino, Consiglio Nazionale delle Ricerche (IAS—CNR), Campobello di Mazara, 91021 Trapani, Italy

**Keywords:** cytisine, buccal film, transmucosal drug delivery, drug-membrane interaction, permeability, mucoadhesion, permeation enhancer, ex vivo model

## Abstract

Cytisine (CYT) is a powerful anti-smoking compound which could greatly benefit from transbuccal delivery because of both its unfavorable pharmacokinetics after oral administration and its intrinsic ability to permeate the buccal mucosa. This work aims to design CYT-loaded buccal thin films suitable for transbuccal drug delivery due to its capability of promoting the interaction between CYT and the buccal membrane. The solvent casting method was employed to prepare several thin films combining various excipients such as matrixing polymers, mucoadhesion agents, plasticizers and other compounds as humectants and sweeteners, component ratios and solvents. A total of 36 compositions was prepared and four of them emerged as the most promising in terms of aspect and flexibility. They all demonstrated homogeneity, thinness, low swelling degree, and controlled drug release according to the Power Law and Peppas-Sahlin mathematical models. Mainly, they proved able to interact with the ex vivo porcine buccal mucosa producing mucoadhesive effects, and act as potent permeation enhancers. In particular, Film B emerged as suitable as it produced a 10.6-fold *Kp* enhancement and a great *Js* value (52.33 μg/cm^2^·h^−1^), even when compared to highly concentrated CYT solutions.

## 1. Introduction

The oral administration of active substances is not always successful. A valuable and attractive alternative could be represented by transbuccal drug delivery. In comparison to oral administration, the mucosal tissues of the oral cavity have a few unique advantages. Particularly, the buccal mucosa is characterized by high vascularization, decreased enzymatic activity, easy administration, and the possibility of immediate withdrawal of the dosage form on the occurrence of side effects. Additionally, this route of administration offers the possibility of avoiding gastric acid hydrolysis and bypassing the hepatic first pass effect. The latter could result in enhanced bioavailability and reduced undesirable effects by lowering of both the required dose and the number of daily doses. Moreover, due to direct access to the jugular vein, this route is excellent to treat acute conditions which require a rapid clinical response (e.g., craving symptoms). The combination of the mentioned factors contributes to determine high patient adherence and compliance with transbuccal treatments [1,2]. Cytisine (CYT) is a valuable and actual example of powerful molecule that may greatly benefit from the advantage of transbuccal delivery. It is a naturally occurring compound that recently emerged as novel promising therapeutic approach in the treatment of nicotine addiction due to its action as a partial agonist of the nicotinic acetylcholine receptors (nAChRs). Its clinical use has already been approved in central Europe, and tablets/capsules for oral administration (Tabex^®^ and Desmoxan^®^; CYT dose: 1.5 mg per tablet) are already on the market. Unfortunately, CYT pharmacokinetics after oral administration are quite unfavorable (e.g., short half-life; high apparent volume of distribution), leading to a restricted and complicated therapeutic regiment, thus reducing the adherence to therapy [3,4,5,6]. Nevertheless, CYT has already been proven to possess the intrinsic ability to permeate the buccal mucosal membrane, thus emerging as candidate for the design of effective transbuccal delivery systems [7]. The major challenges of the transbuccal route are related to a limited absorption area and to the barrier properties of the buccal mucosa [8]. The key factors affecting the effectiveness of buccal administration are mainly related to the drug characteristics (e.g., partition coefficient, molecular weight, solubility/melting point, ionization, diffusion coefficient) and to the Drug Delivery System (DDS) characteristics (e.g., composition, release behavior, penetration/permeation enhancer properties) [9]. Conventional buccal dosage forms frequently fail against salivary turnover and mechanical stresses derived from masticatory movements [1,10,11]. The winning strategy to guarantee the efficiency of the therapeutic effects may be to propose a comfortable formulation capable of extending the association between the active drug and the membrane mucosal barrier. In this view, mucoadhesive buccal thin films have gained great attention due to extremely high patient acceptance, ease of application, thinness, and deformability concurrence to negligible discomfort. Buccal films generally consist of non-dissolving matrix dosage forms able to control the drug release rate. Furthermore, mucoadhesive films allow an intense and protracted drug-membrane interaction due to their ability to interact and bind with the mucus mucins. The main outcome of the films is improved drug absorption and thus bioavailability due to several abilities: to entrap the drug in amorphous state (which is more soluble and readily absorbable), to prolong the contact time of a high concentrate drug solution with the absorption site, and to directly act as a permeation enhancer. An additional advantage is the ease of scale-up because of the adaptability and practicability of the film manufacturing process, such as solvent casting [1,12]. Based on these considerations, this work aims to design CYT-loaded thin films suitable for transbuccal prolonged drug delivery. By an accurate selection of polymers, mucoadhesive agents, plasticizers, and additives, in appropriate ratios, and by optimization of the preparation method, highly reproducible thin matrix films in terms of thinness, drug content, swelling degree, and kinetic of drug release were obtained. The feasibility of film administration was proven by using porcine buccal mucosae as a valuable ex vivo model to assess the interaction between the formulations and the biological membrane and predict human in vivo behavior of drug from films.

## 2. Materials and Methods

### 2.1. Materials

Cytisine (CYT) was kindly supplied from A.C.E.F. Spa (Fiorenzuola D’Arda, Italy). Polyvinylpyrrolidone K90 (PVP K90) and Polyvinylpyrrolidone Crosslinked (PVP CLM), Propylene Glycol, Xylitol, and Polyethylene glycol 200 and 1000 (PEG200 and PEG1000) were obtained from Farmalabor (Canosa di Puglia, Italy). Polyvinylpyrrolidone K30 (PVP K30) was supplied by Fagron Italia S.r.l. (Bologna, Italy). Eudragit^®^ RS100 [Poly(ethyl acrylate, methyl methacrylate, trimethyl-ammonioethyl methacrylate chloride) 1:2:0.1; average MW: 150 kDa; ammoniomethacrylate units: 4.48–6.77%; residual monomers: max 250 ppm ethyl acrylate and max 50 ppm methyl methacrylate] was purchased from Rofarma (Milan, Italy). Trifluoroacetic Acid (TFA), Polyvinylalcohol (PVA), Triacetin, Triethyl Citrate were obtained from Merk (Darmstadt, Germany). Glycerin was purchased from Carlo Erba Reagents (Milan, Italy). The isotonic saline solution (0.9% *w*/*v*) was prepared by dissolving 9 g of sodium chloride (NaCl) in 1 L of distilled water. The isotonic saline solution containing trehalose (5% *w*/*v*) was prepared by dissolving 9 g of NaCl and 50 g of trehalose in 1 L of distilled water. The simulated saliva (pH 6.8) was prepared by solubilizing NaCl (0.126 g), KCl (0.937 g), NaHCO_3_ (0.631 g), KSCN (0.189 g), KH_2_PO_4_ (0.655 g), urea (0.200 g), Na_2_SO_4_ (0.154 g), NH_4_Cl (0.178 g), and CaCl_2_ (0.130 g) in 1 L of distilled water. Phosphate buffered saline (PBS) (pH 7.4) was prepared by dissolving NaCl (8.00 g), KCl (0.20 g), KH_2_PO_4_ (0.24 g), and Na_2_HPO_4_·2 H_2_O (1.81 g) in 1 L of distilled water. All chemicals and solvents (analytical grade) were purchased from Carlo Erba Reagents (Milan, Italy) and were used without further purification. Porcine buccal tissues were kindly supplied by the Municipal Slaughterhouse of Villabate (Palermo, Italy).

### 2.2. Preparation of CYT-Loaded Buccal Films

CYT-loaded buccal films were prepared by the solvent casting method. According to the compositions reported in Table 1 and Table 2, several mixtures having film components for a total weight of 1 g were prepared by dissolving one by one of each excipient in the appropriate volume of the chosen solvent/solvent mixture. In particular, Eudragit^®^ RS 100 (main matrixing polymer), plasticizers, other additives, mucoadhesive agents, and finally CYT were added in this order under magnetic stirring and at room temperature until a fluid, transparent, and homogeneous viscous solution was formed.

Each solution was then poured into a silicon mold and dried in an oven (StabiliTherm, Thermo Scientific, Canton, MA, USA) at 40 °C and 50% of Relative Humidity for 24 h (methanol solutions) or 72 h (ethanol solutions). The so-formed films were then left to equilibrate at room temperature and humidity for 24 h and checked for any imperfections or air bubbles. From each film, small disks (area 0.38 cm^2^ for further characterizations or 1.33 cm^2^ for mucoadhesion evaluations) were obtained by using a biopsy punch. Samples were stored in polystyrene heat-sealed bags at room temperature in a glass container to maintain the integrity and elasticity of the films. Each composition was prepared in triplicate (*n* = 3).

### 2.3. Folding Endurance

To evaluate the suitability of the prepared buccal films, the best formulations obtained in terms of aspect were subjected to flexibility evaluations which were assessed by folding endurance. Briefly, a specific region of each film was repeatedly folded at the same point until it broke, or was folded to 300 times (end point) without breaking. The number of foldings allowed for each film was reported as its folding endurance value [13]. Each experiment was performed manually by considering two regions (center and edge) of each prepared film (*n* = 6). The best buccal films in terms of both aspect and folding endurance were further studied.

### 2.4. Yield and Uniformity

The buccal films were accurately weighted to calculate the yield of the solvent casting technique as follows:

$$Yield\;\%=\frac{weight\;of\;the\;film\;\left(g\right)}{total\;weight\;of\;the\;whole\;components\;\left(g\right)}\times 100$$

Subsequently, the whole film was cut by a biopsy punch to obtain film disks for further characterizations (0.38 cm^2^). Disks uniformity was assessed in terms of weight variation, thickness, and drug content. The thickness and the weight variation were measured on randomly selected film disks by a digital micrometer (Digital Micrometer DIN 863IP40, ABS-system, 0–25 mm/0–1 inch, Vogel Germany, Kevelaer, Germany) and an analytical five decimal balance (mod. AE 240, Mettler-Toledo S.p.A., Milan, Italy) respectively. CYT content was evaluated on randomly selected film disks by both UV-VIS and HPLC-DAD analyses. In brief, to perform the UV-VIS studies, each disk was transferred into a 20 mL flask, dissolved in methanol by sonication, and then brought to volume with the same solvent. The obtained clear solution was then subjected to UV-VIS measurements by using a Shimadzu 1601 Instrument (Kyoto, Japan). To quantify CYT, the following calibration curve in methanol was constructed: λ_max_ = 309 nm, in the linearity range 0.006–0.020 mg/mL the regression curve was Abs= 0.02025 + 39.08 × [mg/mL] (R = 0.999; SE = 0.005). No interference between CYT and the other components of the formulations was observed at the testing concentrations, and no changes in drug absorbance at its λ_max_ were experienced in the presence of the excipients. Interferences were investigated by preparing and analyzing CYT standard solutions containing the employed excipients in the appropriate ratio. Intraday and interday variations were lower than the sensibility.

To perform the HPLC-DAD analyses, each film disk was dipped into 8 mL of Milli-Q water at room temperature and subjected to 5 min of sonication to extract the loaded amount of CYT. This procedure was repeated 5 times and each liquor was collected, transferred into a 50 mL flask, and filled to volume with Milli-Q water. The CYT amount in the obtained clear solution was then detected by using a HPLC Shimadzu LC-10AD VP Instrument (Tokyo, Japan) equipped with a binary pump LC-10AD VP, a UV SPD-M20A Diode Array Detector, a 20 µL loop injector and a computer integrating apparatus (EZ Start 7.4 software, Shimadzu Scientific Instruments, Inc., Columbia, MD, USA). Chromatographic separation was achieved via a reversed-phase column ACE^®^ EXCEL 5 CN-ES (5 µm, 4.6 × 125 mm; Advanced Chromatography Technologies Ltd., Aberdeen, UK) as a stationary phase, while acetonitrile and TFA 0.1% (*v*/*v*) in Milli-QWater (10:90 *v*/*v*) were used as a mobile phase in isocratic conditions. The flow rate was set at 1 mL/min and the UV-VIS wavelength at 305 nm (observed range 200–700 nm). In these conditions, CYT retention time was 2 min. To construct the calibration curve CYT standard solutions in Milli-Q water were prepared (concentration range: 0.004–0.100 mg/mL) and injected. The regression curve was Area = 5.26 × 10^4^ + 5.11 × 10^7^ [mg/mL]. HPLC reports were highly reproducible and linearly related to concentration (R = 0.999). Results are expressed as *DL*% and *LE*%:

$$DL\%=\frac{CYT\;\left(\mathrm{mg}\right)}{small\;disk\;weight\;\left(\mathrm{mg}\right)}\times 100$$

$$LE\%=\frac{recovered\;CYT\;amount\;\left(\mathrm{mg}\right)}{theoretical\;CYT\;amount\;\left(\mathrm{mg}\right)}\times 100$$

All the yield (*n* = 3) and uniformity data (*n* = 9) are reported as means ± SE. Batches were considered uniform when low standard error values were obtained.

### 2.5. Fourier Transform Infrared Spectroscopy (FTIR) in Attenuated Total Reflectance (ATR) Mode Analysis

FTIR-ATR mode spectra were recorded using a Nicolet iS5 instrument (Thermo Scientific™, Waltham, MA, USA) equipped with a ZnSe ATR unit ID7 (Thermo Scientific™, USA) for surface analysis. Spectra were collected in scans in the 4000–500 cm^−1^ spectral range (32 scans, resolution pair: 2 cm^−1^) and rationed to the appropriate background spectra. The following samples were analyzed: pure CYT, Eudragit^®^ RS100, and CYT-loaded buccal films. To evaluate film homogeneity, the analyses were performed on 3 different randomly selected regions of each film (*n* = 9).

### 2.6. X-ray Diffraction (XRD) Evaluations

To evaluate the CYT physical state when embedded into the buccal films a D-8 Focus X-ray diffractometer (Bruker, Billerica, MA, USA) was used by setting the following parameters: from 5° to 60° in θ/2θ (2°/min), 40 kV voltage, 30 mA current, room temperature. XRD analyses were performed on pure CYT and CYT-loaded films. To evaluate film homogeneity the analyses were performed on 3 different randomly selected regions of each film (*n* = 9).

### 2.7. Swelling Studies

Swelling tests were conducted by placing a dry film disk on a glass support and accurately weighing it. Then, 100 μL of simulated saliva were added to wet the disk every 5 min for 60 min. At each time point, the excess of fluid was gently removed with a filter paper and then the weight of the wet disk was assessed (analytical five decimal balance mod. AE 240, Mettler-Toledo S.p.A., Milan, Italy). The swelling degree (*SwD*%) was calculated as follows:

$$SwD\%=\frac{\left(W_{s}-W_{d}\right)}{W_{d}}\times 100$$

where *W_s_* corresponds to the weight of the swollen sample and *W_d_* represents the weight of the dry sample [14]. Each experiment was performed in triplicate (*n* = 3). Results are reported as means ± SE.

### 2.8. In Vitro Drug Release Studies and Kinetics Evaluations

To better mimic the in vivo conditions of the oral cavity, CYT release behavior was in vitro evaluated by using the flow-through system previously described [15]. To summarize, a constant rate (0.3 mL/min) of pre-warmed (37.0 ± 0.1 °C) simulated saliva (pH 6.8) is forced by a peristaltic pump (uniPERISTALTICPUMP 1, LLG-Labware, Meckenheim, Germany) to a release chamber in which the prepared film disk is located. In these conditions, the salivary fluid layer wetting the formulation was about 0.1 mm thick. At scheduled time intervals (5 min), aliquots (1.5 mL) of solution from the release chamber were collected, and CYT concentration was determined spectrophotometrically, as reported above. The experiments were carried out for 3 h and performed in quadruplicate (*n* = 4). At the end of each experiment, the release chamber was disassembled and the remaining formulation was evaluated in terms of CYT residual amount to verify that the sum of the drug released, and the residual drug matched the drug content. To understand CYT release behavior and the mechanism of drug discharge, these data were curve-fitted to the semi-empirical equations usually applied to evaluate kinetics of drug release from delivery systems by using Origin 8.5 as software [7].

### 2.9. Ex Vivo Mucoadhesion Tests

The ex vivo mucoadhesive strength evaluations were performed using a modified two-armed physical balance [16]. Porcine buccal mucosa excised from just slaughtered pigs was used as model tissue, and before the start of the experiment it was equilibrated in isotonic solution for 1 h at room temperature. Then the tissue was placed on a glass support and kept in a vessel placed in a thermostatic bath at 37.0 ± 0.5 °C maintaining the temperature for the whole experiment. For each test, a film disk (1.33 cm^2^) was fixed at the lower side of a rubber stopper with a bi-adhesive tape on one balance pan. The opposite pan was counterbalanced with a beaker to collect water placed in the other pan of the balance. A plastic tube connected to a peristaltic pump (uniPERISTALTICPUMP 1, LLG-Labware, Meckenheim, Germany) that allows a constant rate of distilled water (0.6 mL/min) was suspended above the beaker. Before starting the measurements, the tissue was wetted with 500 µL of simulated saliva and then the tested formulation was placed in close contact to the mucosal surface by applying a light force with a weight of 20 g. Mucoadhesion measurements started after different preload times (5, 10, 15, 20 and 30 min) to evaluate the mucoadhesive behavior. At the end of each preload time the balance and the peristaltic pump were activated. The test continued until the complete detachment of the formulation from the mucosal surface occurred. Then the weight of the water collected in the beaker was accurately measured (analytical five decimal balance mod. AE 240, Mettler-Toledo S.p.A., Milan, Italy) and the adhesive strength, expressed as the mass (g) required to detach the formulation from the mucosal surface was calculated as follows:

$$Adhesive\;Strength\;\left(N\right)=g\times \frac{9.81}{1000}$$

Then the Detachment Force was calculated by normalizing the obtained adhesive force (N) to the formulation surface (expressed in m^2^):

$$Detachment\;Force\;\left(\frac{N\;}{m^{2}}\right)=\frac{Adhesive\;Strength\;\left(N\right)}{Formulation\;Surface\;\left(m^{2}\right)}$$

Each experiment was performed in triplicate (*n* = 3). Results are expressed as means ± SE.

### 2.10. Ex Vivo Permeation/Penetration Studies through Porcine Buccal Mucosa

#### 2.10.1. Tissue Preparation

Porcine buccal specimens were collected from the vestibular area of the retro molar trigone of freshly slaughtered domestic, 6–8-month-old pigs intended for human consumption (no ethical approval required). Tissue samples were accumulated immediately after animal slaughter and transferred within 1 h to the laboratory in refrigerated boxes. After washing in isotonic solution, the excess tissue was removed and then the specimens were stored at −80 °C for at least one week (pre-treatment for 1 h with a trehalose-containing isotonic solution 5% *w*/*v* as a cryoprotectant). Immediately before the ex vivo studies, the tissue was equilibrated at room temperature, washed for 1 h in isotonic solution, and then the buccal mucosa membrane was obtained by thermal shock by briefly dipping the tissue in pre-warmed isotonic solution (≈1 min; 60.0 ± 0.5 °C) and then carefully peeling off the mucosa from the adipose tissue and connective (slides 250 ± 25 μm thick, evaluated by a digital micrometer VWR International, Milan, Italy) [17,18,19].

#### 2.10.2. Permeation Assay

Vertical Franz-type diffusion cells (Permeagear, flat flange joint, 9-mm orifice diameter, 15 mL acceptor volume, SES GmbH—Analysesysteme, Bechenheim, Germany) were used as a two-compartment open model. First, the previously obtained buccal mucosa was equilibrated in isotonic solution overnight at room temperature to remove all the biological matter that could interfere with drug quantification. Then, Franz cells were mounted using adequate sections of the mucosa (as a membrane) between the acceptor and the donor chambers, filled with PBS (pH 7.4) and simulated saliva (pH 6.8) respectively and left to equilibrate at 37.0 ± 0.5 °C for 15 min. Afterwards, the donor fluid was removed and replaced with a film disk (0.38 cm^2^) soaked with 500 μL of simulated saliva. Aliquots (500 μL) of acceptor fluid were withdrawn at scheduled time intervals, replaced with fresh medium, and subjected to UV-Vis analyses by using a Shimadzu 1601 Instrument (Kyoto, Japan). The following calibration curve was constructed: λ_max_ = 305 nm; preparation of CYT standard solution in PBS pH 7.4; linearity range 0.0006–0.024 mg/mL, regression curve Abs = 0.0052 + 39.13 [mg/mL] (R = 0.999; SE = 0.009). Nor interference, neither relevant interday-intraday variations affected the measurements. Each experiment was repeated six times (*n* = 6) and performed at 37 ± 0.5 °C for 6 h under continuous magnetic stirring. At the end of the experiments each film disk was subjected to visual and tactile inspections to verify its integrity. Furthermore, the donor medium was withdrawn, centrifuged, and spectrophotometrically analyzed to verify the actual CYT concentration in the donor chamber as this is a useful value for further mathematical elaborations.

#### 2.10.3. Entrapment Studies

After the permeation assays, the Franz cell were disassembled, and the mucosal membranes were collected and washed with distilled water (2 mL) to remove any drug residue on the surface. Then, the amount of CYT entrapped into the buccal mucosa was extracted by warm methanol (55.0 ± 5.0 °C, 2 mL) for 2 min twice. The aliquots were collected in a 10 mL flask, brought to volume and quantified by UV-Vis measurements by construction the following calibration curve: λ_max_ = 309 nm, in the linearity range 0.0005–0.008 mg/mL, the regression curve was Abs = 0.04342 + 41.15 × [mg/mL] (R = 0.999; SE = 0.005).

#### 2.10.4. Biopharmaceutical Parameters

Data were further examined to calculate the following biopharmaceutical parameters: *Js*, *Kp*, *t_lag_* and *De*.

Drug flux (*Js*) through the buccal mucosa was calculated at the steady state per unit area by linear regression analysis of permeation data according to the following equation:

$$Js=\frac{Q}{A\times t}\left(\frac{\mu g}{{\mathrm{cm}}^{2}\times h}\right)$$

where *Q* is the amount of CYT (μg) permeated during the time interval *t* (h) and *A* is the area of the buccal mucosa available for permeation (0.636 cm^2^). At the steady state, *Js* is equal to the slope of the obtained straight line. The constant of permeability (*Kp*) was then calculated by the relationship:

$$Kp=\frac{Js}{Cd}\left(\frac{\mathrm{cm}}{h}\right)$$

where *Cd* is the experimental CYT concentration detected into the donor compartment at the end of the permeation assay.

The *t_lag_* (min) was determined from the interception with the x axis of the obtained straight line at the steady state.

Similarly, at the end of each experiment, the amount of drug entrapped (*De*) per unit area into the mucosal tissue was calculated using the following relationship:

$$De=\frac{Q_{T}}{A}\left(\frac{\mu g}{{\mathrm{cm}}^{2}}\right)$$

where *Q_T_* is the amount (μg) of CYT entrapped into the tissue and *A* is the area available for accumulation (0.636 cm^2^) [15,20,21].

Origin 8.5 software was used for mathematical data processing and results are expressed as means ± SE.

### 2.11. Data Analysis

Data are expressed as mean ± standard error (SE). All differences were statistically evaluated by one-way analysis of variance (ANOVA or F-test) with the minimum level of significance set at *p* < 0.05.

## 3. Results and Discussion

### 3.1. Design, Preparation and Screening of CYT-Loaded Buccal Films

One common and effective strategy of drug delivery is the entrapment of the active substance into a matrix system. Among the various matrix systems, thin films are quite suitable for buccal application. They have been widely proposed for the latter application as an effective alternative to conventional dosage forms, as they lead to increased drug retention on the target site, and consequently, enhanced bioavailability while being easily administrable and patient friendly. Indeed, buccal thin films are solid, manageable, stable, flexible, and resistant dosage forms which could be accurately designed to display a reproducible and controlled drug release pattern as well as promoted ability to strongly interact with the mucosal tissue thus resulting in suitable muco-adhesiveness and permeation/penetration enhancer effect.

As CYT has already been proved to be able to cross the buccal membranes [7], the possibility of enhancing CYT-membrane interaction thus leading to relevant drug fluxes and significant increase in terms of *Kp* by effective buccal films is here exploited.

Generally, buccal films consist of an ingredient combination including matrixing polymers, mucoadhesive agents, plasticizers, and other additives selected to confer specific properties (e.g., permeation enhancers, humectants) or promote patients’ compliance (e.g., sweetener). However, some excipients could possess a variety of characteristics which make it difficult to catalogue. Other key factors which directly affect the resulting buccal film are related to the preparation method, such as the employed solvent and its volume, as well as the drying conditions when the solvent casting process was used. In this work Eudragit^®^ RS100 was selected as the main matrixing polymer. It is polymethacrylate derivative was chosen because of its ability to form matrix structures suitable for controlled drug release, limited swelling degree and muco-adhesiveness. It is characterized by a pH-independent behavior that makes it further suitable for buccal administration, as it is not susceptible to environmental pH variations related to smoking, food and beverage ingestion, or clinical conditions [22,23]. In addition, several mucoadhesive agents (PVA, PVP K90, PVP K30, PVP CLM), plasticizers (Glycerin, Triacetin, Triethyl Citrate, Propylene Glycol, PEG200 and PEG1000) and other excipients (Sorbitol, Xylitol) were tested at various ratios to obtain buccal films able to load the 5% (*w*/*w*) of CYT and suitable in terms of both aspect and deformability. A total of 36 formulations was evaluated: EtOH-based from FE-1 to FE-18; MeOH-based from FM-1 to FM-18 as reported in the materials and methods section. Indeed, the choice of the solvent used to disperse all the ingredients is crucial for the success of a homogeneous matrix system after drying. The solvent must be able to solubilize all the ingredients in a limited volume, resulting in clear and uniform solutions, while it also must have a good volatility for the drying process to be fast enough.

Ethanol (EtOH) was firstly chosen as it is non-toxic. However, while during the preparation of the starting solution, all the mucoadhesive agents employed seemed to completely dissolve; they precipitated during the drying process leading to unsatisfactory final products characterized by large white plaques. As a consequence, the best EtOH-based formulations in terms of final aspect were those prepared in absence of mucoadhesive additives (from FE-13 to FE-18).

In contrast, Methanol (MeOH) led to easily insert the mucoadhesive agent PVP K90, although small amounts of water had to be added to it to completely solubilize xylitol (when present) achieving great results in terms of buccal films appearance. Even though MeOH is a toxic solvent, it was employed as the following preparation method was already proven to produce non-toxic buccal film thus suggesting the complete removal of the solvent [24].

To prepare the buccal films the solvent casting method was employed, and the required drying conditions (temperature and time) were carefully evaluated and related to the employed solvent. To establish solvent casting parameters, preliminary tests were conducted. As a result, temperatures over 40 °C led to rapid solvent evaporation (both EtOH and MeOH), allowing bubble formation and roughness on the film surface. When MeOH was used as solvent, a drying step protracted over 24 h leads to brittle and stiff films. In contrast, the buccal films prepared using EtOH required 72 h to allow complete solvent evaporation. The 96% (*v*/*v*) EtOH used for film preparation is an azeotrope mixture, and multistep evaporation rates occur mainly due to water uptake, leading to a prolonged evaporation and drying time respecting the MeOH-based solutions [25].

Together with the aspect evaluations, the folding endurance of the prepared buccal films after equilibration with the environmental condition was monitored, to collect information regarding their flexibility and eventual fragility. The deformability of the formulations depends both on the employed plasticizer(s) and its/their amount/ratio and on the humectants eventually inserted which could attract the surrounding water during the environmental equilibration step which follows preparation. Among the several prepared films most, of them were thus discharged due to lack of flexibility. FE-17, FE-18, FM-17 and FE-18 emerged as they achieved the fixed end point without breaking, confirming good deformability and ability to withstand the imposed mechanical stress, as desired [26]. This is a quite relevant parameter as deformable films could better adapt to the mucosal surface of application thus being comfortable.

Table 3 reports the best film compositions and their denomination for subsequent studies.

The best obtained buccal films were prepared by employing the following excipients: Eudragit^®^ RS100; PVP-K90 (selected for its muco-adhesiveness) [24,27,28]; propylene glycol (selected as plasticizer to confer flexibility, deformability and softness as well as due to its antibacterial properties) [28,29]; PEG1000 (selected because of its film-forming ability, capability to promote the water uptake acting as a plasticizer and promoting CYT release as well as for its muco-adhesiveness) [30,31] and xylitol (selected as permeation enhancer and low-calory sweetener also for diabetic patients) [32].

**Figure 1:**
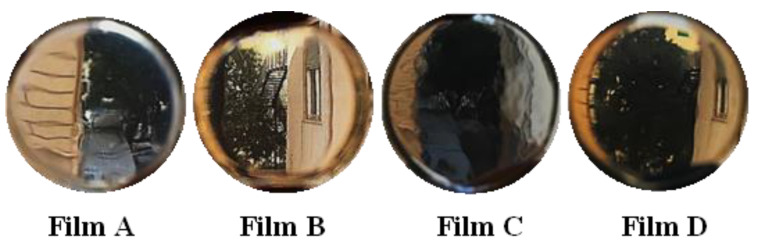
Appearance of the freshly prepared CYT-loaded Buccal Films.

The reported four different compositions led to flexible, smooth, transparent, and homogeneous films (Figure 1). 

### 3.2. Characterization of the Most Promising CYT-Loaded Buccal Films

The performance of the preparation method was assessed in terms of yield percent and reproducibility evaluations by measuring the average weight, thickness, and drug content of randomly selected 0.38 cm^2^ film disks. Data are reported in Table 4 and prove the reproducibility and the suitability of the solvent casting method as well as the homogeneity of each sample, as the calculated standard error values are quite narrow. Additionally, the quantitative evaluations of CYT were performed by both UV-Vis (cheaper, quicker, and easier method) and HPLC-DAD analyses. The latter were performed to assure drug integrity: neither degradation peaks nor modification in terms of shape, retention time, or new peaks were detected in the spectral range 200–700 nm, confirming that the preparation method does not affect CYT chemical structure. Moreover, it is worth emphasizing that the buccal films here proposed are very thin (0.44–0.59 mm thick), and this is an additional factor that can contribute to increased patient compliance.

**Figure 2:**
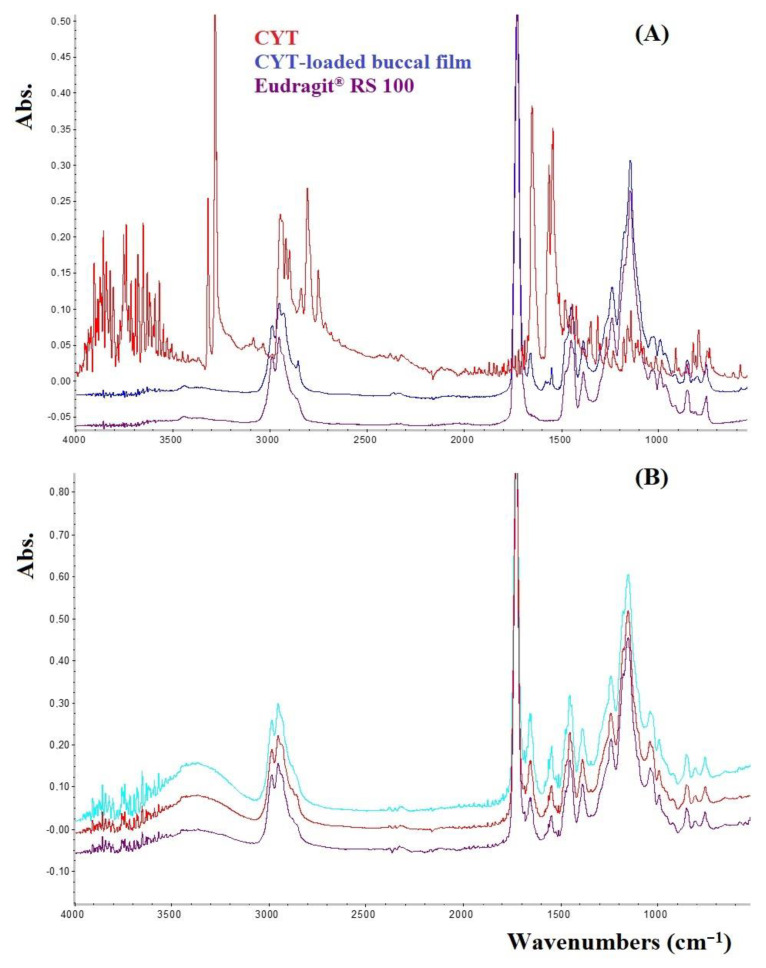
(**A**) FTIR in ATR mode spectra of pure CYT (red), Eudragit^®^ RS100 (purple) and Film B (blue) reported as a representative example for all the prepared film. (**B**) FTIR in ATR mode spectra recorded by analyzing different three portions of Film B.

To increase the knowledge on the prepared formulations, both FTIR and XRD studies were carried out. According to the literature data [7], the FTIR spectra (Figure 2A) still confirm the existence of such interactions between CYT and Eudragit^®^ RS 100 during the solvent casting process. This is highlighted by the disappearance of CYT bands at 3278 and 3319 cm^−1^ when observing the buccal films’ spectra. These bands are related to the NH group of CYT which could be subjected to rotamerization producing two distinct bands (PubChem). Their disappearance therefore suggests that the NH group is involved in the interaction with the polymer [33]. As the Eudragit-CYT physical mixture (data not shown) resulted in the sum of the two single spectra, it is conceivable that the occurring drug-polymer interaction is due to the preparation method: the interaction might arise in solution and due to the gradual solvent evaporation it did not break. In addition, similar spectra were obtained by analyzing different portions of the same film (Figure 2B) thus further confirming sample homogeneity.

**Figure 3:**
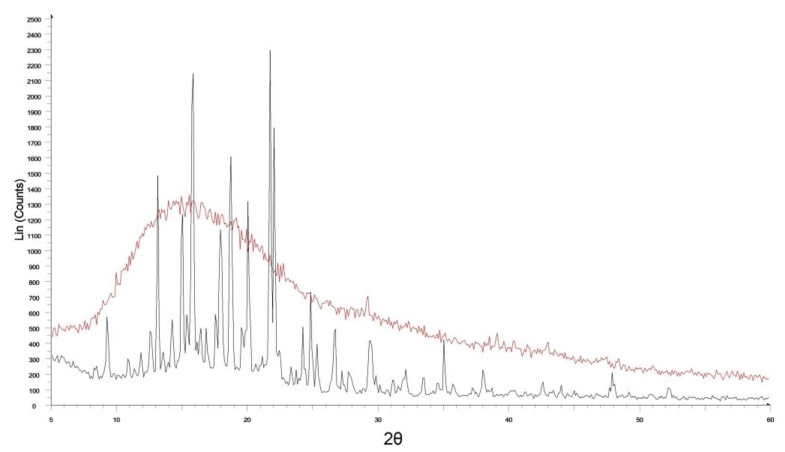
X-ray diffraction patterns of pure CYT (black) and Film B (red) reported as a representative example for all the prepared film.

The XRD analysis gives information regarding the drug physical state (amorphous or crystalline) when embedded into the solid matrix. The XRD evaluations (Figure 3), confirm the absence of crystalline molecules. This is due to the complete amorphization of CYT and xylitol (when present) during the film preparation, as expected and desired. The obtainment of CYT amorphous form could be crucial and suitable, as this physical state is characterized by enhanced solubility thus promoting several key processes (e.g., dissolution, drug discharge and permeation).

**Figure 4:**
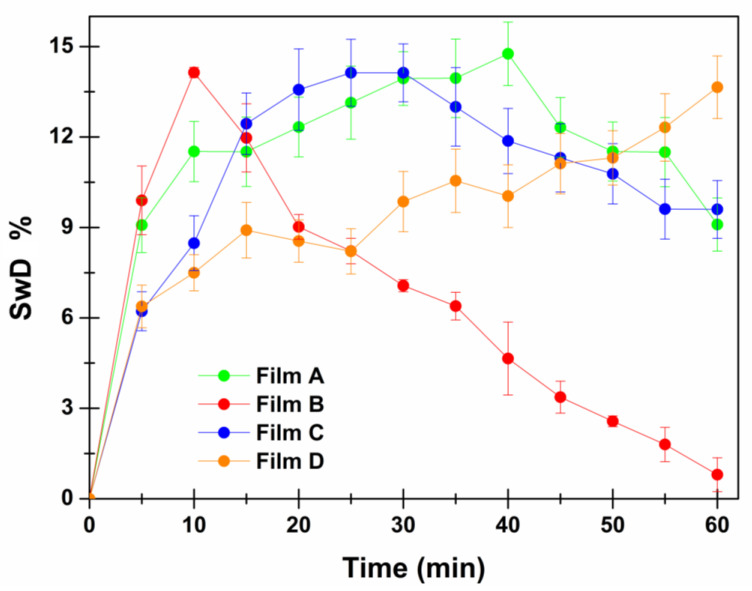
Swelling Degree % as a function of time (min) for Film A (green), Film B (red), Film C (blue), Film D (orange). Data are reported as means ± SE (*n* = 3).

For buccal administration purposes the swelling degree is a relevant parameter as it provides interesting information about the predictability in vivo compliance following the application of the device. Indeed, the proposed buccal films are designed to be places middle third in the mediodistal direction and caudal third in the cranio-caudal direction, in an area less involved in mandibular movements and less subjected to salivary leaching. Here, if they absorb high amount of water from the saliva (high swelling degree) they could cause discomfort thus reducing patients’ compliance [34]. The swelling degree data are reported in Table 5 and graphically presented in Figure 4 as percentage of weight increase referred to the starting weight. All the formulations showed a slight swelling aptitude (maximum observed value ≤ 15%), while the swelling behaviors resulted different. Generally, Film A, B, and C showed a trend of starting weight increase followed by a descendant patter (maximum observed values: 14.76 ± 1.05% at 40 min, 14.14 ± 0.17% at 10 min and 14.13 ± 1.11% at 25 min for Film A, B, and C respectively). Film D is the only one that shows a different swelling behavior, characterized by an increase of the swelling degree overtime and a maximum value of 13.65 ± 1.04% at the last considered time point (60 min). The experimental data suggest that the presence of PVP K90 modify the polymer meshes slowing down the water uptake process. On the other hand, the presence of xylitol maximizes and accelerates water uptake, consequently modifying the swelling behavior. Indeed, Film B and Film C (both characterized by the presence of xylitol) reached their maximum swelling degree % faster than Film A (no xylitol, no PVP K90), while Film C (presence of PVP K90) displayed a slower pattern that Film B (absence of PVP K 90). Finally, Film D (presence of PVP K90; absence of xylitol) was the only one characterized by a gradual increasing trend with no detectable maximum. It is also important to highlight that the apparent reduction of the swelling degree % observed for Film A, B and C after the maximum value could be due to dissolution of the water-soluble components from the formulation (both excipients and drug).

**Figure 5:**
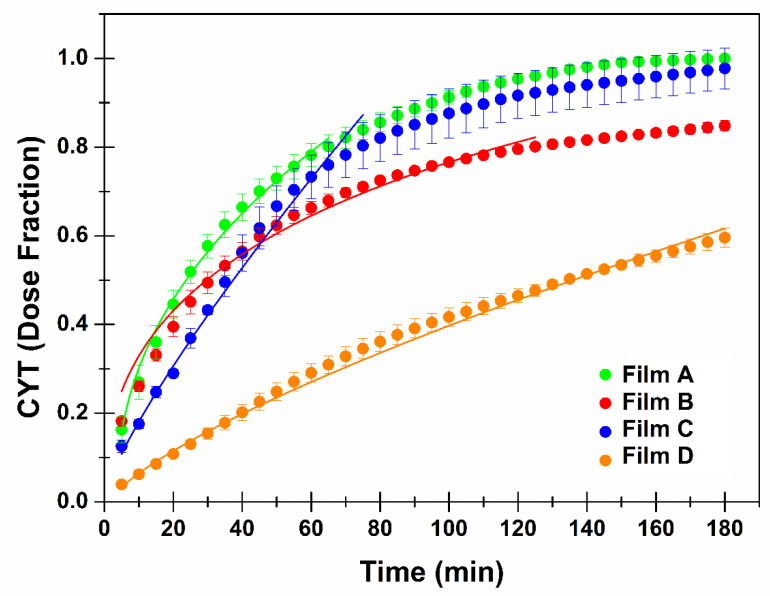
CYT release pattern from the buccal films: dose fraction of CYT released as a function time (min). Continue lines represent the best fitting curve: Power Law (considering the T_lag_) for Film A (green) e D (orange); Peppas-Sahlin for Film B (red) and C (blue). Values are presented as means SE ± (*n* = 4).

To produce drug transport though the target tissue, the proposed buccal films should be able to release CYT to the biological fluid. Generally, to evaluate drug release behavior, the pharmacopoeias describe experimental conditions which employ high dissolution media volume (sink or pseudo-sink conditions). However, for buccal administration purposes, it is better to mimic the in vivo conditions which involve small amount of liquid (saliva) and sink conditions not forced. As a consequence, the CYT release experiments from the prepared buccal films were conducted by using a peristaltic pump connected to a release chamber capable of continuously licking the formulation with a thin layer of salivary fluid. Furthermore, the experimental release data (Figure 5) were curve fitted by the most common mathematical models used in this field in order to identify the main kinetic governing the release pattern [35]. 

The fitting studies (Table 6) highlighted that the excipient composition, and particularly the presence/absence of xylitol, directly affects the mechanism of CYT release. In particular, the best fitting results were obtained applying the Power Law (Film A and D; no xylitol) and the Peppas–Sahlin (Film B and C; presence of xylitol) equations. Moreover, the release rate data are in accordance with the swelling data. Indeed, it is confirmed that PVP K90 limited the water entry thus slowing down both the swelling phenomenon and the drug release rate (Film D).

The Korsmeyer-Peppas (also named Power Law) model is usually applied to describe drug release from polymeric systems when the discharge mechanism is unknown when multiple processes occur. The experimental *n* value gives information about the release mechanism: *n* = 0.5 Fickian model (diffusion); 0.5 < *n* < 1 non-Fickian anomalous transport (diffusion and swelling), *n* = 1 non-Fickian model (swelling and relaxation); *n* > 1 non-Fickian Super Case II model (relaxation and breaking of the polymeric chains) [37]. In particular, the calculated *n* values for Film A and D were 0.443 and 0.739 respectively. As a consequence, CYT release from Film A resulted mainly related to a Fickian diffusional process. This is probably due to Eudragit^®^ RS100 which is the main component of the formulation. In contrast, the CYT release mechanism from Film D is governed by both diffusion and swelling as the presence of PVP K90 modify the whole polymeric structure leading to a time-dependent and gradual swelling: as the water entry increases by time, the swelling phenomenon contribution became relevant over time thus explaining the obtained *n* value.

The Peppas-Sahlin equation deriving from the power law ones aims at distinguishing the contribution of both the diffusion and relaxation processes. To obtain this information the Relaxation (R) and Diffusion (D) parameters should be calculated by the following equations [36]:

$$F=\frac{1}{1+\frac{k_{2}}{k_{1}}\;\cdot \;t^{m}}$$

$$\frac{R}{F}=\frac{k_{2}\;\cdot \;t^{m}}{k_{1}}$$

**Figure 6:**
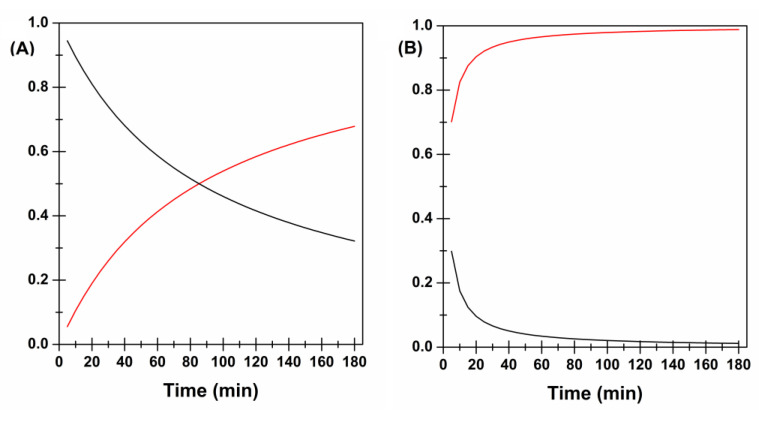
Diffusion (F–black) and Relaxation (R-red) contributions in the CYT release process from Film B (**A**) and Film C (**B**) as a function of time.

Figure 6 graphically presents the *F* and *R* behavior over time for Film B (panel A) and Film C (panel B).

Film B initially displayed a release mechanism mainly governed by diffusion. However, after 80 min of experiment, a trend reversal was observed, and the relaxation contribution became dominant. This is probably due to xylitol (and other water-soluble components) dissolution which over time lead to the creation of larger pores thus “relaxing” the whole structure.

As already discussed, the insertion of PVP K90 may modify the whole polymer chain disposition, causing a variation in terms of matrix meshes, water uptake, and release pattern. According to the swelling data, PVP K90 decreases the water entry rate thus hindering and slowing down drug diffusion through polymer chains. To permit a massive water uptake, PVP K90 chains must relax, causing a modification of the whole structure and consequently the solubilization of water-soluble molecules that lead to a further relaxation. As a consequence, the relaxation process appears to be the predominant one. Furthermore, it should be noted that the effects of PVP K90 were not observed in FILM C as the influence of xylitol was predominant.

### 3.3. Interaction between CYT-Loaded Buccal Films and the Ex Vivo Porcine Mucosal Tissue

**Figure 7:**
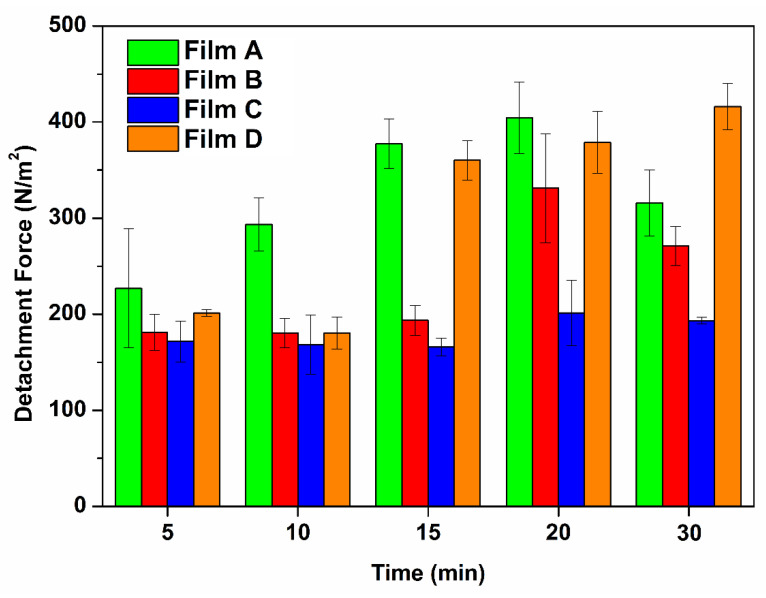
Detachment Force (N/m^2^) as function of the contact preload time (min) with the buccal mucosa when evaluating Film A (green), Film B (red), Film C (blue), Film D (orange). Values are presented as means ± SE (*n* = 3).

The terms mucoadhesion or bio-adhesion indicate the adhesion of the drug delivery system to the mucosal membrane epithelium. When the drug is intended to be administered through the transmucosal routes (e.g., when administered by a buccal thin film) increasing the residence time of the formulation in the mouth can potentially help to enhance bioavailability and absorption rate. The mucoadhesion phenomenon is a complex process which depends on the interaction between the application site and formulation. Due to its complexity, mucoadhesion could be explained by several theories (wetting, fracture, diffusion-interlocking, electronic, adsorption, and dehydration theories). Generally, it is schematized in two main stages: the contact stage and the consolidation stage. During the contact stage, the drug delivery system approaches the mucosa, and here it is wetted. The main factors affecting the contact stage are the formulation capability of hydrating and its ability to spread the interfacial forces between the material and the mucosa. In the consolidation stage, various physicochemical interactions between the formulation, the mucus layer, and the mucosal membrane take place (physical entanglement as well as chemical bonds) resulting in a prolonged adhesion [38]. The mucoadhesion evaluations were conducted by using the method of the two-arms modified balance. The latter consists of the evaluation of the mass (g) required to completely detach the formulation from an ex vivo mucosal tissue soaked with a small volume of simulated saliva in order to mimic the in vivo conditions. Subsequently, the obtained mass could be converted into mucoadhesion strength (related to the contact area) and detachment force (independent from the contact area). Porcine buccal fresh specimen was selected as ex vivo tissue to be employed as it has extensively been proved to display high similarity with human oral epithelium and thus it represents a valuable membrane model [39]. As reported in Table 7 and also graphically presented in Figure 7, generally the proposed buccal films exhibited muco-adhesiveness as a function of preload contact time. It is likely to notice that both Film A and Film B formulations exhibited a gradually increase of the mucoadhesive strength together with the increase of the starting contact time, reaching a maximum after 20 min of preload contact time and then falling down. However, while Film A mucoadhesion interestingly increases at each considered time point until 20 min, for Film B the obtained results at 5, 10 and 15 min were quite comparable. Film C resulted the less mucoadhesive one and highlighted a pattern which does not significantly vary by increasing the preload time. Finally, the force of adhesion calculated for Film D gradually grew while increasing the contact time for the whole considered time points. The muco-adhesiveness of the chosen polymers (Eudragit^®^ RS100, PEG1000 and PVP K90) appears evident when observing the behavior of Film A and Film D. Additionally, it is clear that the presence of xylitol negatively affected the muco-adhesiveness of the whole system (Film B < Film A) and this is much more evident in presence of PVP K90 (Film C < Film D). This could be attributable to xylitol dissolution, and consequently both to the formation of a xylitol-enriched salivary solution on the mucosal surface which may alter and/or reduce the contact surface as well as to the xylitol-induced tissue modifications (as it is a permeation enhancer which transitory alter the tissue integrity). 

A formulation able to strongly interact with the mucosal surface of application could lead to a promoted drug-tissue interaction, resulting in enhanced drug absorption and consequently bioavailability. To verify the permeation enhancer ability of the proposed buccal film, ex vivo transbuccal permeation experiments were performed. To this aim, Franz type vertical diffusion cells were chosen as a two-compartments open model able to mimic the in vivo condition of a buccal administration (e.g., small donor volume, large acceptor volume). In addition, porcine buccal mucosa was employed as mucosal barrier interposed between the donor and the acceptor chambers, due to its already mentioned high similarity with human oral tissues. Indeed, the literature extensively reports the histomorphology similarity between human and porcine specimens and consequently the possibility of employing the porcine buccal mucosa to predict human buccal absorption [40].

**Figure 8:**
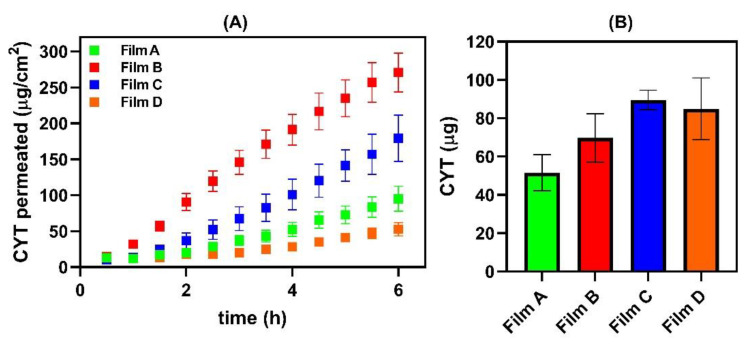
(**A**) CYT (mg/cm^2^) permeated as a function of time (h). (**B**) Amount (μg) of CYT accumulated into the buccal membrane (0.636 cm^2^) after 6 h of experiment applying Film A (green), Film B (red), Film C (blue) and Film D (orange). Results are presented as means ± SE (*n* = 6).

CYT permeation profiles from the proposed buccal films are reported in Figure 8A, while the amount of drug accumulated into the buccal mucosa at the end of each ex vivo experiment is shown in Figure 8B.

As expected, the presence of xylitol (Film B and C) positively affected the permeation profiles, producing higher drug fluxes than those observable for the xylitol-free films. In contrast, the accumulation into the tissue is higher in presence of PVP K90.

To evaluate the actual ability of the proposed films to promote CYT interaction with the buccal mucosal membrane, the experimental data were further elaborated to calculate the biopharmaceutical parameters reported in Table 8. Additionally, the real CYT concentration into the donor chamber at the end of the ex vivo tests was experimentally evaluated and employed to correctly calculate the Kp and Ac parameters. It should be pointed out that by applying a film disk (0.38 cm^2^), the CYT dose initially inserted into the donor chamber is 2 mg/mL (≈1 mg of CYT per disk, soaked with 500 μL of simulated saliva), and that the experimental CYT concentration into the saliva recovered from the donor chamber at the end of the experiments was ≈ 1 mg/mL for all the reported buccal films. This suggest that each formulation created a CYT-enriched solution whose concentration is dependent on the experimental conditions and independent of the release behavior. To understand the relevance of the extrapolated parameters they were compared to the following previously published data [7]:

- The application of a 2 mg/mL CYT solution into the donor chamber in the same experimental conditions produced a drug flux (*Js*) of 4.94 µg/cm^2^∙h^−1^;
- The maximum CYT flux was obtained by applying a 10 mg/mL CYT solution (46.77 µg/cm^2^∙h^−1^), whereas higher concentrations did not produce an increase in flux;
- CYT *Kp*, when evaluating a 2 mg/mL solution, was underestimated as CYT amount was too low to observe its permeability;
- CYT *Kp*, when evaluating a 10 mg/mL solution, was truthful and resulted in 0.00468 cm/h.

In this review, it is shown that all designed formulations are able to improve CYT permeability through porcine buccal mucosa when compared to CYT alone administered as a solution. This is confirmed by significantly observed *Kp* enhancement, which generally increases by an order of magnitude. Even the worst formulation (Film D) allowed a 2.2-fold *Kp* enhancement, while a significant 10.6-fold increase is observable for the best one (Film B). Moreover, a significant enhancement in terms of CYT flux is observable by administering the Film B formulation; the obtained flux is 10.6-fold higher compared to that observed by administering a 2 mg/mL solution, and even slightly higher than the maximum *Js* obtainable by applying a 10 mg/mL solution. These data strongly suggest the capability of the proposed buccal films to interact with the mucosal membrane and modify its permeability, thus acting as potent permeation enhancers. Furthermore, it is noticeable that the obtained *De* values (which represent the amount of CYT entrapped into the buccal tissue per unit area after 6 h of experiment) were lower for all the buccal films compared to the reference CYT solutions. This further confirms the actual occurrence of modification of the tissue, resulting in decreased drug retention while maximized drug permeation. This is also in accordance with the experimental *t_lag_* which were strongly reduced by the buccal films. 

It is evident that with an accurate choice of both the components and the preparation method, it is possible to obtain a CYT-loaded thin film able to interact with the mucosal ex vivo membrane, thus positively influencing drug biopharmaceutical parameters and bioavailability.

These results could be extended to other drugs and may represent support for the development of new transmucosal delivery systems.

## 4. Conclusions

The main scope of this work was to embed CYT into matrix buccal thin films to propose comfortable and effective formulations that are able to exploit CYTs intrinsic ability to permeate the buccal mucosa and improve the CYT-mucosa interaction, resulting in a permeation enhancer effect. The solvent casting method was chosen as a convenient, easy, rapid, and scalable technique, and the main relevant parameters were appropriately evaluated. Both ethanol and methanol were evaluated as the starting solution preparation, and once the main matrixing polymer was selected (Eudragit^®^ RS100), several other excipients (mucoadhesion agents, plasticizers, other additives) were tested in various ratios to produce buccal films with a fixed CYT content (5% *w*/*w*). While EtOH failed to produce buccal films including the selected mucoadhesion agents, MeOH resulted great to incorporate PVP K90. Among the 36 prepared formulations (FE 1–18 in EtOH and FM 1–18 in MeOH) four of them emerged for their homogeneous and transparent aspect, as well as for their good flexibility. The best formulations, subsequently named Film A, B, C, and D, were further characterized. They all displayed uniformity, (in terms of drug content and weight), thinness, and low swelling degree (≤14%). The FTIR analysis in ATR mode confirmed the occurrence of CYT-Eudragit interactions, as well as sample homogeneity. Additionally, the XRD evaluations highlighted the total amorphization of each crystalline compound (CYT and xylitol when present). The key point of this work is in the ex vivo experiments, which demonstrated the ability of the proposed buccal films to interact with porcine buccal mucosal membranes (chosen as a valuable model for human buccal tissue), producing mucoadhesive interactions and significantly enhancing CYT flux thought the tissue. Among all the formulations, Film B displayed the best ex vivo results. The latter displayed an extremely high CYT flux (higher than the maximum flux obtainable by evaluating CYT solutions at superior concentrations) and a 10.6-fold increase of *Kp*. Its ability as a penetration enhancer is probably related to its interaction with the tissue, resulting in membrane modifications. This is further confirmed by the reduction in the amount of the drug which remains entrapped into the specimen at the end of the ex vivo test, as well as by the significant *t_lag_* reduction.

To summarize, accurate excipient selection together with the definition of the main key points of the preparation method could lead to suitable CYT-loaded buccal thin films, supporting the ability of innovative transbuccal delivery systems to interact with and opportunely modify biological membranes and positively influence drug permeation and, consequently, bioavailability.

## Figures and Tables

**Table 1:** Weight composition (mg) of CYT-loaded buccal films prepared by solvent casting method by using ethanol (mL) as solvent.

		Mucoadhesion Agents				Plasticizers					Others			
Formula Code	Eudragit^®^ RS100	PVP K90	PVP K30	PVP CLM	PVA	GL	TRC	PG	PEG200	PEG1000	SOR	XYL	CYT	EtOH
**FE-1**	790	60	-	-	-	100	-	-	-	-	-	-	50	15
**FE-2**	790	60	-	-	-	50	50	-	-	-	-	-	50	12.5
**FE-3**	740	-	-	-	60	100	50	-	-	-	-	-	50	20
**FE-4**	540	-	260	-	-	-	50	100	-	-	-	-	50	12
**FE-5**	500	-	260	-	-	-	50	100	-	-	40	-	50	12
**FE-6**	550	-	260	-	-	-	-	100	-	-	40	-	50	12
**FE-7**	400	-	410	-	-	-	-	100	-	-	40	-	50	10
**FE-8**	550	-	-	260	-	-	-	100	-	-	40	-	50	10
**FE-9**	638	-	-	-	212	-	-	100	-	-	-	-	50	10
**FE-10**	750	60	-	-	-	-	-	100	-	-	40	-	50	15
**FE-11**	750	40	-	-	-	-	-	100	60	-	-	-	50	15
**FE-12**	750	-	40	-	-	-	-	100	60	-	-	-	50	16
**FE-13**	750	-	-	-	-	-	-	100	100	-	-	-	50	10
**FE-14**	425	-	-	-	-	-	-	100	-	425	-	-	50	10
**FE-15**	567	-	-	-	-	-	-	100	-	283	-	-	50	10
**FE-16**	638	-	-	-	-	-	-	100	-	212	-	-	50	10
**FE-17**	750	-	-	-	-	-	-	100	-	100	-	-	50	12
**FE-18**	700	-	-	-	-	-	-	100	-	100	-	50	50	12

GL: Glycerin, TRC: Triacetin, PG: Propylene Glycol, SOR: Sorbitol, XYL: Xylitol.

**Table 2:** Weight composition (mg) of CYT-loaded buccal films prepared by solvent casting method by using methanol (mL) as solvent.

		Mucoadhesive Agent	Plasticizers						Others			
Formula Code	Eudragit^®^ RS100	PVP K90	GL	TRC	TC	PG	PEG200	PEG1000	SOR	XYL	CYT	MeOH
**FM-1**	790	60	100	-	-	-	-	-	-	-	50	9
**FM-2**	740	60	150	-	-	-	-	-	-	-	50	8
**FM-3**	740	60	100	-	50	-	-	-	-	-	50	17.5
**FM-4**	740	60	100	50	-	-	-	-	-	-	50	17.5 + 0.2 mL acetic acid
**FM-5**	740	60	50	100	-	-	-	-	-	-	50	15
**FM-6**	740	60	100	50	-	-	-	-	-	-	50	12
**FM-7**	740	20	120	70	-	-	-	-	-	-	50	15
**FM-8**	740	60	100	50	-	-	-	-	-	-	50	20
**FM-9**	790	60	50	50	-	-	-	-	-	-	50	7.5
**FM-10**	790	60	-	100	-	-	-	-	-	-	50	9
**FM-11**	590	60	-	100	-	-	200	-	-	-	50	9
**FM-12**	590	60	-	100	-	200	-	-	-	-	50	6
**FM-13**	690	60	-	100	-	100	-	-	-	-	50	9
**FM-14**	790	60	-	50	-	50	-	-	-	-	50	5
**FM-15**	740	60	-	-	-	150	-	-	-	-	50	7
**FM-16**	570	100	-	-	-	200	-	-	80	-	50	7
**FM-17**	650	50	-	-	-	100	-	100	-	50	50	23 + 2 mL H_2_O
**FM-18**	700	50	-	-	-	100	-	100	-		50	23 + 2 mL H_2_O

GL: Glycerin, TRC: Triacetin, TC: Triethyl Citrate, PG: Propylene Glycol, SOR: Sorbitol, XYL: Xylitol.

**Table 3:** Weight composition (mg) and denomination of the best resulting CYT-loaded buccal films.

Formula Code	Denomination	Eudragit^®^ RS100	PVP K90	Propylene Glycol	PEG1000	Xylitol	Cytisine	Solvent(s)
**FE-17**	**Film A**	750	-	100	100	-	50	12 mL EtOH
**FE-18**	**Film B**	700	-	100	100	50	50	12 mL EtOH
**FM-17**	**Film C**	650	50	100	100	50	50	23 mL MeOH + 2 mL H_2_O
**FM-18**	**Film D**	700	50	100	100	-	50	23 mL MeOH + 2 mL H_2_O

**Table 4:** Characterization of the CYT-loaded buccal films: yield % of the solvent casting method (*n* = 3), weight (mg), thickness (mm), CYT content (mg), drug loading % (DL%) and loading efficacy % (LE%) of a 0.38 cm^2^ disk ± SE (*n* = 9).

Formulation	Yield (%)	Weight (mg)	Thickness (mm)	CYT Content (mg)	DL% *	LE %
**Film A**	93.9 ± 2.1	15.56 ± 1.09	0.59 ± 0.04	0.75 ± 0.05	4.82 ± 0.14	96.48 ± 2.83
**Film B**	95.8 ± 1.8	17.87 ± 0.42	0.48 ± 0.04	0.84 ± 0.02	4.71 ± 0.13	94.27 ± 2.57
**Film C**	95.8 ± 2.2	17.35 ± 1.17	0.48 ± 0.04	0.96 ± 0.06	5.53 ± 0.15	110.60 ± 3.00
**Film D**	94.9 ± 1.6	18.12 ± 0.33	0.44 ± 0.08	0.83 ± 0.02	4.60 ± 0.10	92.00 ± 2.00

* Quantitative CYT measurements by UV-Vis and HPLC-DAD analyses gave superimposable results.

**Table 5:** Swelling Degree % data reported as means ± SE (*n* = 3).

Swelling Degree %				
Time (min)	Film A	Film B	Film C	Film D
**5**	9.08 ± 0.91	9.90 ± 1.14	6.22 ± 0.65	6.38 ± 0.71
**10**	11.52 ± 1.00	14.14 ± 0.17	8.48 ± 0.91	5.50 ± 0.60
**15**	11.51 ± 1.15	11.97 ± 1.13	12.44 ± 1.02	8.91 ±0.92
**20**	12.33 ± 0.99	9.02 ± 0.41	13.57 ± 1.35	8.55 ± 0.70
**25**	13.14 ± 1.21	8.22 ± 0.42	14.13 ± 1.11	8.21 ± 0.75
**30**	13.94 ± 0.89	7.07 ± 0.20	14.13 ± 0.96	9.86 ± 1.00
**35**	13.95 ± 1.30	6.39 ± 0.46	13.00 ± 1.30	10.55 ± 1.05
**40**	14.76 ± 1.05	4.65 ± 1.21	11.87 ± 1.08	10.04 ± 1.04
**45**	12.32 ± 0.99	3.37 ± 0.59	11.31 ± 1.13	11.12 ± 1.00
**50**	11.52 ± 0.98	0.57 ± 0.18	10.78 ± 1.00	11.31 ± 0.90
**55**	11.50 ± 1.15	1.80 ± 0.57	9.61 ± 0.99	12.32 ± 1.12
**60**	9.10 ± 0.88	0.80 ± 0.56	9.60 ± 0.96	13.65 ± 1.04

**Table 6:** Mathematical models fitted to the experimental release data: calculated fitting parameters and square of correlation coefficient (R^2^). Data were fitted until the 80% of CYT released (Film A, Film B and Film C, respectively) or the 60% of CYT released (Film D).

Applied Mathematical Model	Film A	Film B	Film C	Film D
**Zero Order** **M_t_/M_∞_ = k × t**	k = 0.0147 ± 0.0008 R^2^ = 0.655	k = 0.0077 ± 0.0001 R^2^ = 0.472	k = 0.0138 ± 0.0005 R^2^ = 0.917	k = 0.0036 ± 0.0001 R^2^ = 0.9942
**First Order** **M_t_/M_∞_ = F_MAX_ × (1 − e ^−k × t^)**	k = 0.0272 ± 0.0005 R^2^ = 0.986	k = 0.0232 ± 0.0002 R^2^ = 0.975	k = 0.0190 ± 0.0004 R^2^ = 0.979	k = 0.0052 ± 0.0001 R^2^ = 0.9990
**Higuchi** **M_t_/M_∞_ = k × t^0.5^**	k = 0.1013 ± 0.0011 R^2^ = 0.981	k = 0.0766 ± 0.0002 R^2^ = 0.942	k = 0.0742 ± 0.0033 R^2^ = 0.861	k = 0.0435 ± 0.0002 R^2^ = 0.9720
**Korsmeyer-Peppas (Power Law)** **M_t_/M_∞_ = k × t^n^**	k = 0.0951 ± 0.0097 *n* = 0.516 ± 0.026 R^2^ = 0.980	k = 0.1295 ±0.0138 *n* = 0.386 ± 0.023 R^2^ = 0.969	k = 0.0296 ± 0.0024 *n* = 0.781 ± 0.023 R^2^ = 0.988	k = 0.0109 ± 0.0004 *n* = 0.778 ± 0.008 R^2^ = 0.9996
**Korsmeyer-Peppas (Power Law)** **considering T_lag_** **M_t_/M_∞_ = k × (t − T_lag_)^n^**	k = 0.1324 ± 0.0112 T_lag_ = 3.58 ± 0.48 *n* = 0.443 ± 0.021 R^2^ = 0.993	k = 0.3745 ± 0.1705 T_lag_ = 25.87 ± 20.42 *n* = 0.166 ± 0.097 R^2^ = 0.848	k = 0.0249 ± 0.0065 T_lag_ = -1.24 ± 1.75 *n* = 0.821 ± 0.061 R^2^ = 0.988	k = 0.0134 ± 0.0012 T_lag_ = 1.59 ± 0.59 *n* = 0.739 ± 0.017 R^2^ = 0.9997
**Hixson-Crowell** **M_t_/M_∞_ = 1 × [1 − (1 − k × t)^3^]**	k = 0.0074 ± 0.0003 R^2^ = 0.935	k = 0.0038 ± 0.0001 R^2^ = 0.853	k = 0.0058 ± 0.0001 R^2^ = 0.985	k = 0.0015 ± 0.0001 R^2^ = 0.9983
**Peppas-Sahlin** **M_t_/M_∞_ = k_1_ × t^m^ + k_2_ × t^2m^** (m = 0.43) *	k_1_ = 0.0984 ± 0.0104 k_2_ = 0.0067 ± 0.0020 R^2^ = 0.977	k_1_ = 0.1321 ± 0.0067 k_2_ =-0.0036 ± 0.0009 R^2^ = 0.979	k_1_ = 0.0170 ± 0.0050 k_2_ = 0.0186 ± 0.0012 R^2^ = 0.989	k_1_ = 0.0070 ± 0.0009 k_2_ = 0.0065 ± 0.0001 R^2^ = 0.9995

* The m value was determined as reported in the literature [36] by calculating the aspect ratio of each formulation (diameter/thickness).

**Table 7:** Force of Adhesion and Detachment Force of CYT-loaded buccal films as function of time contact with mucosa. Values are presented as means ± SE.

Contact Preload Time (min)		Formulation			
		Film A	Film B	Film C	Film D
**5**	N	0.0302 ± 0.0082	0.0241 ± 0.0025	0.0228 ± 0.0028	0.0268 ± 0.0005
	N/m^2^	226.85 ± 61.92	181.01 ± 18.82	171.64 ± 21.07	201.36 ± 3.69
**10**	N	0.0390 ± 0.0037	0.0240 ± 0.0020	0.0224 ± 0.0041	0.0240 ± 0.0022
	N/m^2^	293.44 ± 27.77	180.47 ± 15.05	168.12 ± 30.61	180.38 ± 16.63
**15**	N	0.0502 ± 0.0034	0.0258 ± 0.0021	0.0221 ± 0.0013	0.0479 ± 0.0027
	N/m^2^	377.43 ± 25.53	193.59 ± 15.57	165.89 ± 9.44	360.28 ± 20.54
**20**	N	0.0538 ± 0.0050	0.0441 ± 0.0076	0.0268 ± 0.0045	0.0504 ± 0.0043
	N/m^2^	404.50 ± 37.32	331.25 ± 56.89	201.27 ± 33.93	378.76 ± 32.45
**30**	N	0.0420 ± 0.0046	0.0361 ± 0.0027	0.0257 ± 0.0004	0.0553 ± 0.0032
	N/m^2^	315.73 ± 34.19	271.10 ± 20.54	193.36 ± 3.28	416.10 ± 24.04

N: Force of Adhesion—N/m^2^: Detachment Force.

**Table 8:** Extrapolated CYT biopharmaceutical parameters (*Js*, *Kp*, *t_lag_*, and *De* ± *SE*) and experimental concentration into the donor chamber when evaluating the buccal films as CYT solutions as a reference.

Sample	*Js* (µg/cm^2^∙h^−1^)	*Kp* (cm/h)	*De* (µg/cm^2^)	[CYT]_DONOR_ (mg/mL)	Lag Time (min)
**Film A**	18.61 ± 3.23	0.01918 ± 0.00465	81.03 ± 70.80	0.97 ± 0.12	59
**Film B**	52.33 ± 6.45	0.04961 ± 0.00611	109.59 ± 19.89	1.06 ± 0.15	17
**Film C**	35.70 ± 5.97	0.03143 ± 0.06780	140.78 ± 8.03	1.14 ± 0.20	64
**Film D**	10.40 ± 2.60	0.01040 ± 0.00230	133.65 ± 40.97	0.81 ± 0.11	NO
CYT 2 mg/mL solution *	4.94 ± 0.16	0.00247 ± 0.00001	164.72 ± 13.12	2.00	NO
CYT 10 mg/mL solution *	46.77 ± 6.18	0.00468 ± 0.00062	559.30 ± 93.66	10.00	100

* Literature data [7].

## Data Availability

The data presented in this study are available on request from the corresponding author.
